# Faster, Smarter, Precise: Integrating Speed Breeding and CRISPR-Based New Genomic Techniques into the Conventional Field Crop Breeding Pipeline

**DOI:** 10.3390/plants15152389

**Published:** 2026-08-04

**Authors:** Velimir Mladenov, Rada Šućur, Borislav Banjac, Milana Čurčić, Teodora Feher Kričković, Jasna Musić, Sofija Petrović, Bojan Drašković, Faheem Shehzad Baloch, Wei Hu

**Affiliations:** 1 PanCrop Lab, Faculty of Agriculture, University of Novi Sad, Trg Dositeja Obradovića 8, 21000 Novi Sad, Serbia; rada.sucur@polj.edu.rs (R.Š.); borislav.banjac@polj.edu.rs (B.B.); milana.curcic@polj.edu.rs (M.Č.); teodora.feher@polj.edu.rs (T.F.K.); jasna.music@polj.edu.rs (J.M.); sofija.petrovic@polj.edu.rs (S.P.); 2 KWS Srbija, DOO, 22300 Stara Pazova, Serbia; bojan.draskovic@kws.com; 3 Department of Biotechnology, Faculty of Science, Mersin University, Yenişehir, 33343 Mersin, Türkiye; faheem.baloch@mersin.edu.tr; 4 Tropical Crops Genetic Resources Institute, Chinese Academy of Tropical Agricultural Sciences, Haikou 571101, China; huwei.catas@gmail.com

**Keywords:** speed breeding, generation advancement, new genomic techniques, CRISPR, plant breeding, genomic selection

## Abstract

Generation time is the principal bottleneck constraining genetic gain in plant breeding. Speed breeding (SB) addresses this by simultaneously managing the photoperiod, light spectrum and intensity, temperature, CO_2_ concentration, mineral nutrition, growing substrate volume, and post-harvest seed dormancy, enabling four to seven generations per year in long-day cereals and legumes, and four to five generations in optimised short-day systems. This review evaluates SB against the conventional pedigree framework; synthesises validated environmental parameters and crop-specific protocols; and examines principal SB applications in hybridisation, genomic selection, disease-resistance screening, and allele introgression. A structured comparison of major review and protocol papers identifies broad consensus on core parameters alongside genuine divergence on far-red light supplementation. Critical evaluation addresses genotype-by-environment interaction, incomplete trait coverage, infrastructure costs, and unresolved questions on the biological integrity of rapidly advanced generations. The review further discusses new genomic techniques (NGTs), particularly CRISPR/Cas9-based gene editing, in the context of the EU’s June 2026 NGT regulation, under which Category 1 plants carrying only targeted endogenous modifications are exempt from GMO authorisation. When combined with SB, this regulatory shift can compress the interval from gene-editing event to registered variety from decades to a few years. Speed breeding, NGTs, and conventional field-based selection are most productively treated as complementary elements of a unified pipeline.

## 1. Introduction

With the global population on course to reach nine to ten billion people by mid-century, food production must increase by an estimated 60–80 percent—a challenge compounded by accelerating climate change and the inherently slow pace of conventional crop improvement [1,2,3]. The rate at which new cultivars can be developed is not primarily limited by a shortage of useful genetic diversity, but rather by the time needed to convert that diversity into fully homozygous, agronomically stable lines suitable for field evaluation. In self-pollinating species such as wheat, this process involves four to six rounds of self-pollination after the initial cross, each round confined to one growing season. Once multi-environment testing and seed multiplication are included, the total duration from cross to registered variety routinely spans ten to fifteen years [4,5]. This generation time constraint is now intersecting with a shifting regulatory landscape in Europe: on 17 June 2026, the European Parliament gave final approval to the new genomic technique (NGT) regulation, which establishes a simplified genetically modified organism (GMO)-exempt pathway for Category 1 plants deemed equivalent to conventionally bred varieties [6].

Because annual genetic gain is inversely proportional to breeding cycle length for a given selection intensity and selection accuracy, shortening the years required per generation can markedly increase the rate of gain achievable per calendar year; speed breeding has been reported to translate this relationship into practice, for example, by shortening a wheat breeding cycle from twelve to seven years when combined with genomic selection.

Speed breeding (SB) is a controlled-environment generation advancement technique that compresses the vegetative and reproductive phases of the plant life cycle by simultaneously manipulating photoperiod, light quality and intensity, temperature, and post-harvest seed handling, allowing several generations to be completed within a single calendar year rather than one. Its principal advantage is a substantial reduction in the time required to reach a fully homozygous, evaluable line, which shortens the interval between the initial cross and cultivar release. Its principal limitations are the need for dedicated controlled-environment infrastructure, the restricted set of agronomically important traits that can be reliably expressed or selected under artificial conditions, and a strongly genotype-dependent efficacy. These aspects are examined in detail in Section 2.2 and Section 2.3.

Plant breeders have long sought to reduce this generational delay through a range of approaches including off-season greenhouse multiplication, shuttle breeding between geographically separated growing environments, embryo rescue, and doubled haploid (DH) technology. Each approach has distinct advantages and drawbacks: shuttle breeding can double the annual number of generations through coordinated international field cycles but is still restricted to two seasons per year; DH technology produces fully homozygous lines in one to one and a half years, yet remains poorly responsive in a number of commercially important crops; and embryo culture is both labour-intensive and strongly genotype-dependent.

Among the most recently developed strategies for accelerating pure line development is speed breeding. Its conceptual roots lie in NASA-funded research on plant cultivation under extended artificial photoperiods in closed growth systems, which was subsequently adapted and formalised for field crop breeding by a research team at the University of Queensland, who introduced the term “speed breeding” in 2003 [7,8]. The approach reduces the vegetative duration of each generation by deploying an extended photoperiod—most commonly 20–22 h of daily light—in combination with a spectrally tailored light source, precise temperature control, and the deliberate early harvest of immature seed followed by dormancy-breaking treatments to enable immediate re-sowing. Large-scale proof-of-concept was established by Watson et al. [4], who reported attainment of six generations per year in spring wheat, barley, chickpea and pea, and four generations per year in rapeseed.

Since those landmark publications in 2018, SB methodology has been extended to a growing range of crop families. Initial work focussed on long-day cereals and legumes; purpose-designed LED-based protocols subsequently made SB applicable to short-day and day-neutral species—soybean, rice, amaranth, hemp and cowpea—for which the long-photoperiod approach is counterproductive because it delays or suppresses floral induction [9,10]. Concurrently, SB has been combined with marker-assisted selection (MAS), genomic selection (GS), and genome-editing technologies, allowing breeders to shorten not only the time needed to fix homozygous lines but also the time required to move a target allele through a segregating background or to phenotype large populations for disease resistance [11,12].

A substantial number of reviews have already synthesised aspects of SB technology [5,7,8,13]. The aim of the present review is threefold: (i) to set out the conventional pedigree breeding pipeline as the methodological and temporal baseline against which SB is assessed; (ii) to synthesise the validated technical parameters and crop-specific protocols that define current SB practice, drawing together and comparing the positions of the principal contributors to this literature; and (iii) to provide a critical assessment of the conditions under which SB delivers on its promise of accelerated genetic gain, and the conditions—genotype × environment interaction, incomplete trait coverage, infrastructure cost and unresolved questions of biological validity—under which it may not. What distinguishes the present review is its explicit integration of this established SB literature with the European Union’s newly finalised (17 June 2026) NGT regulatory framework, and its structured, source-by-source cross-tabulation of parameter agreement and divergence across the principal SB studies (Table 1), a comparative synthesis of this kind and this timeliness not undertaken by earlier, single-technology-focused reviews.

## 2. Results and Discussion

### 2.1. Conventional Breeding: Methodology and Constraints

The pedigree method, along with its principal variants—bulk selection, single seed descent (SSD) and backcross breeding—constitutes the methodological reference point against which any accelerated breeding platform must be judged. In temperate cereal crops, a typical programme begins with the assembly of parental germplasm and initial crossing, advances through four to eight generations of inbreeding to fix segregating progeny (F_1_ to F_8_), and culminates in multi-year, multi-environment yield trials before a variety can be released. Every inbreeding cycle is anchored to one field season; the vernalisation requirements of winter cereals, the photoperiod sensitivity of many genotypes, and post-harvest seed dormancy all constitute biological constraints that cannot be circumvented simply by intensifying effort within a season [5,8]. When all stages are summed, the crossing-to-inbreeding phase alone consumes three to seven years, post-inbreeding multi-environment evaluation adds a further four to five years, and seed increase and official registration require one to three more years, placing the total duration well beyond a decade for most annual crops and considerably longer for biennials and perennials [5,15].

A core strength of conventional breeding lies in the fact that selection takes place in the same environment where the variety will ultimately be grown. Exposure to field-level abiotic and biotic stresses—water deficit, pathogen inoculum, soil variability, and ambient temperature fluctuation—creates selection pressure that is directly predictive of agronomic performance, and germplasm selected under these conditions accumulates robustness to environmental variability that is inherently difficult to reproduce in a controlled setting. This is not an incidental limitation; it is the core rationale for why accelerated controlled-environment selection cannot fully displace multi-environment field evaluation.

Conventional breeding programmes are correspondingly either land-, labour- and/or infrastructure-intensive, with costs distributed across seasons rather than concentrated in capital equipment. This structure has direct implications for national breeding programmes in lower-income agricultural systems, where conventional, field-based breeding remains the primary—and often the only practically accessible—route to variety development [7].

### 2.2. Speed Breeding: System, Principles and Validated Protocols

**Figure 1:**
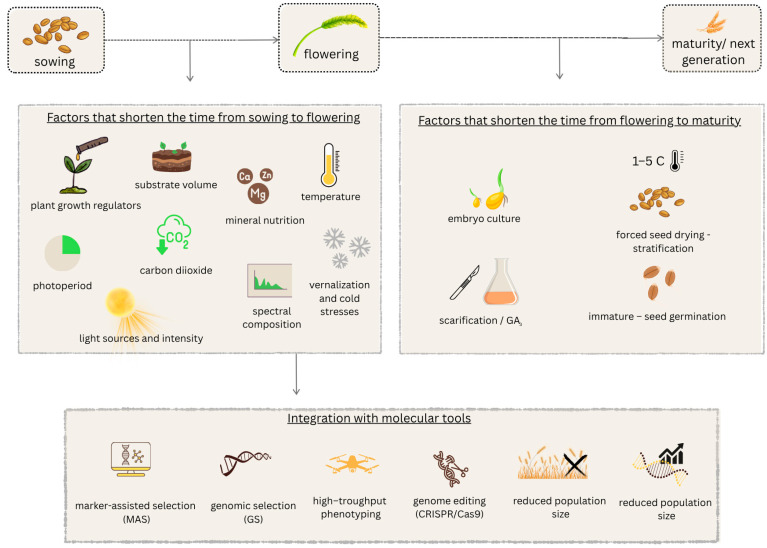
Factors influencing speed breeding across the plant life cycle. The greatest acceleration is achieved not by a single factor in isolation but by an optimised, crop-specific combination of multiple factors.

Rather than a single discrete intervention, speed breeding is more accurately described as a multi-factor acceleration platform in which several simultaneously adjusted environmental variables operate across three distinct phases of the plant life cycle: the pre-flowering vegetative period, the generative period from anthesis to seed maturity, and the post-harvest handling phase in which seed dormancy must be overcome to allow immediate re-sowing. Figure 1 illustrates the key factors and the phase of development at which each exerts its primary effect.

In long-day and day-neutral crops, a daily photoperiod of approximately 22 h of light followed by 2 h of darkness represents the most widely validated regime; under these conditions, flowering initiation is substantially advanced in Triticeae cereals, pea, and rapeseed compared with standard or shorter photoperiods. A brief dark interval is deliberately preserved rather than switching to continuous illumination, because uninterrupted light disrupts circadian rhythm-regulated gene expression and negatively affects overall plant health and development. Short-day crops do not share a single optimal photoperiod: in pigeon pea, an 8 h light/16 h dark cycle is most effective; rice, soybean and cowpea respond best to 10 h photoperiods; hemp and pepper require 12 h cycles; and applying photoperiods that are too short can itself provoke stress-related physiological responses that counterproductively delay flowering onset.

Light quality interacts with photoperiod in crop-specific ways. Jähne et al. [9] demonstrated that an LED-controlled, blue-light-enriched and far-red-deprived spectrum under a 10 h photoperiod produced short, sturdy soybean plants that flowered approximately 23 days after sowing and matured within 77 days, supporting up to five generations per year; far-red light had no effect on soybean flowering time but produced undesirable petiole elongation and lodging, whereas the same far-red supplementation advanced flowering in rice and amaranth by approximately 8 and 3 days, respectively. This crop-specific, sometimes contradictory response to far-red light is echoed in triticale, rapeseed, pepper, amaranth and rice, where far-red light has been shown to elevate transcription of flowering locus T homologues and accelerate the transition to the reproductive phase, while having no effect in pea or soybean [5,16].

Temperature regimes of 22–25 °C during the day are typical for long-day cereals and Brassica species, while warm-season crops such as rice, cotton and soybean tolerate and may benefit from daytime temperatures of 28–32 °C [5]. Pandey et al. [2] report a closely comparable consensus specification of 22 °C day/17 °C night with 70 percent humidity and light intensities of 360–650 μmol m^−2^ s^−1^, while noting that these parameters require adjustment according to species and developmental stage.

Shortening the generative phase draws on two complementary approaches. In embryo culture, developing embryos are excised from the plant approximately two weeks post-anthesis and maintained on a nutrient medium; the rescued embryos develop into transplantable seedlings within 10–20 days. In the forced drying approach, whole immature seeds harvested 10–20 days after flowering are oven-dried at temperatures between 28 and 50 °C for one to seven days, releasing seed from dormancy while avoiding the labour demands and contamination risks of in vitro embryo rescue. When seeds do not germinate spontaneously after drying, additional dormancy-breaking treatments—gibberellic acid (GA) application, cold stratification, mechanical scarification or brief high-temperature exposure—can be applied individually or in combination. In soybean, germination success of forced-dried immature seeds has been shown to rise from around 50 percent at 56 days after sowing to approximately 90 percent at 77 days [9].

#### 2.2.1. Generational Throughput and Validated Crops

**Figure 2:**
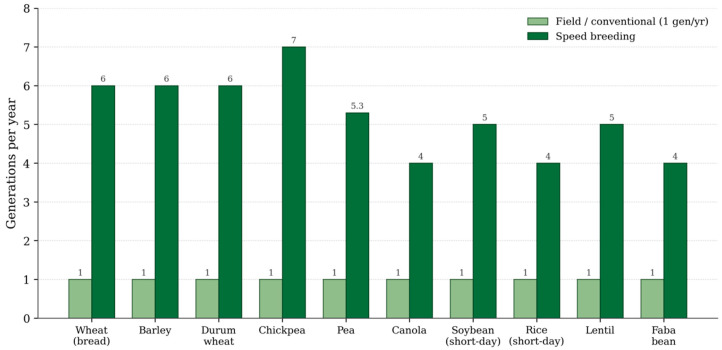
Reported generations per year under field/conventional cultivation versus speed breeding for ten crop species. Values compiled from Blinkov et al. [5], Watson et al. [4], Jähne et al. [9], Samineni et al. [17], and Mobini and Warkentin [18].

Across the literature surveyed, documented generational output under speed breeding ranges from four to seven generations per year in long-day cereals and cool-season legumes, compared with a single generation per year under field conditions and two to three generations per year using winter nurseries or shuttle breeding. Figure 2 summarises the reported generational throughput for ten representative crops drawn from the sources reviewed here.

Table 1 sets these findings in comparative perspective against the conclusions reported by eight major speed breeding reviews and primary protocol papers. The table highlights both convergence—broad agreement that 22 h photoperiods benefit long-day crops and that short-day crops require crop-specific, shorter photoperiods—and divergence, particularly regarding the universality of far-red light supplementation and the magnitude of achievable generational gain in legumes.

#### 2.2.2. Integration with Molecular Tools

The contribution of SB to annual genetic gain is substantially amplified when it operates in conjunction with MAS or GS. Growing large segregating populations through a full accelerated cycle allows genotyping to be carried out early, and seedlings that lack a target allele can be removed before any further growing space or resources are committed to them; the common use of 96-well trays for seedling cultivation allows direct transfer to 96-well genotyping plates and minimises manual handling [5,9]. The combined SB–GS framework is examined in greatest depth by Pandey et al. [2], who describe ‘Speed GS’ schemes in which candidate parents are ranked by their genomic estimated breeding values (GEBVs) and the selected progeny are then advanced under SB conditions, an approach validated in spring wheat and reported to reduce inbreeding depression compared with purely phenotypic parent selection.

Documented application cases illustrate the range of this integration. Rana et al. [19] combined SNP marker-assisted selection with SB to introgress the hst1 salt-tolerance gene into a high-yielding rice background, while Aydin et al. [20] and Cha et al. [21] used a comparable strategy to transfer the Glu-B1i glutenin subunit into bread wheat for improved gluten quality. Disease resistance screening under SB shows a particularly strong correlation with field-based phenotyping: leaf rust response in wheat grown under SB correlated with field measurements at R^2^ = 0.77 [22], and Fusarium head blight (FHB) severity showed a Pearson correlation of r = 0.921 between SB and field conditions [23]. Genome editing pipelines benefit similarly: barley embryos developed under SB conditions show morphogenetic potential and transformation efficiency comparable to conventionally grown plants, shortening the interval between transformation and the phenotypic characterisation of edited lines [4].

### 2.3. Critical Assessment of Speed Breeding

#### 2.3.1. Genotype × Environment Interaction

Plants developed under optimised, uniform, artificial conditions are, by construction, not exposed to the abiotic and biotic stress variation that drives adaptive differentiation in field populations. The risk is that SB selects genotypes that perform well in growth chambers but have not been tested against the environmental heterogeneity they will encounter in farmers’ fields. The evidence on this point is mixed rather than uniformly reassuring. On the one hand, several quantitative traits show a strong, validated correlation between SB and field expression—plant height, growth rate, presence of awns and leaf wax coating, and pod shattering in rapeseed all transfer well [4,5]. On the other hand, Cha et al. [24] report no correlation between field and SB performance for wheat awn length and spikelet number per spike, and SB cannot replicate the G × E variability that field multi-environment trials are specifically designed to sample. Yield itself, and resistance to abiotic stresses, are explicitly identified across multiple sources as traits that cannot be adequately evaluated without field testing [4,5].

#### 2.3.2. Trait Coverage and Phenotypic Fidelity

Not all traits of agronomic importance are accessible to SB selection. Traits dependent on field-level canopy architecture, competitive ability, soil–water dynamics under natural rainfall variability, or exposure to a multi-pathogen, multi-pest field environment may be poorly expressed, or not expressed at all, in growth chambers. The traits that do transfer well are predominantly morphological and developmental (plant height, growth rate, presence of awns or leaf wax, stem colour in hemp) rather than the integrative physiological traits, such as yield components under water limitation, that ultimately determine a variety’s commercial value. This distinction matters for how SB is deployed: it is well suited to qualitative, monogenic or oligogenic traits and to early-generation culling, but a defensible breeding programme cannot substitute SB-based phenotyping for field evaluation of complex, polygenic, environmentally sensitive traits [2,4].

#### 2.3.3. Infrastructure, Cost, and Equity

Speed breeding requires substantial capital investment in LED lighting infrastructure and climate-controlled growth chambers, together with ongoing operational costs; more than half of the total cost of running an SB protocol has been attributed to lighting and temperature control alone [8]. This creates a structural asymmetry: SB is most accessible to well-resourced private and public breeding programmes in high-income countries, while national programmes in lower-income agricultural systems—which serve the majority of the world’s smallholder farmers—frequently have neither the capital infrastructure nor the stable electricity supply needed to deploy it at scale [7]. Potts et al. [7] document this constraint in detail, noting that many African national agricultural budgets allocate as little as three percent of total spending to agriculture, and propose intermediate-technology adaptations—repurposed shipping containers fitted with solar-powered lighting and temperature control—as one route toward more equitable access.

#### 2.3.4. Biological Validity of Accelerated Generations

Compressing the plant life cycle raises legitimate, and not yet fully resolved, questions about whether the resulting plants are biologically equivalent to their field-grown counterparts. Seed harvested at the immature, forced-dried stage shows consistently lower germination rates, delayed sprouting, and reduced productivity in the resulting plants relative to conventionally matured seed [4,14,25]. Plants regenerated from embryo culture likewise show lower productivity than seed-grown plants [26], and green, immature seed used directly for germination in chickpea and soybean is associated with weakened seedling establishment and increased pod shattering [17]. Whether epigenetic stability is maintained across rapid successive generations, and whether stress-response gene networks remain adequately heritable under artificial, low-stress-variance conditions, are not fully resolved in the literature and are identified here as open questions warranting dedicated study.

#### 2.3.5. Integration, Not Replacement

**Figure 3:**
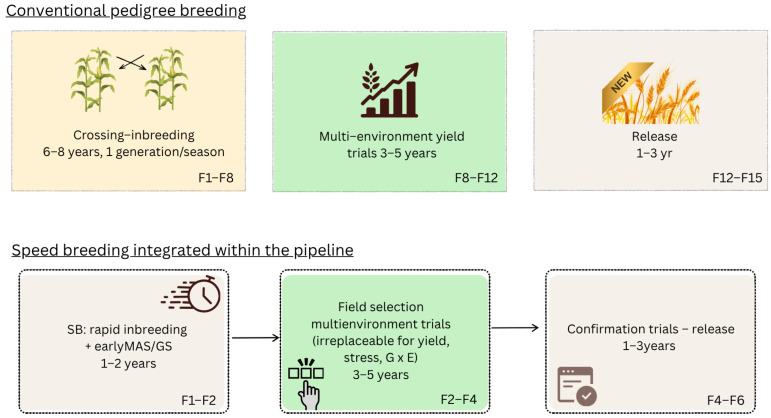
Proposed integration of speed breeding within the conventional breeding pipeline. Speed breeding compresses the crossing-to-inbreeding phase and supports early MAS or GS; field-based multi-environment trials remain irreplaceable for yield, abiotic stress tolerance and other traits subject to strong genotype × environment interaction.

The most coherent conclusion to emerge from this review is that speed breeding should be understood as one stage within a broader breeding programme rather than as an alternative to it. SB delivers the greatest return when applied to early-generation selection, the rapid movement of qualitative or oligogenic traits into elite backgrounds, and the production of large segregating populations for use as genomic training sets—all functions that precede, and supply material to, the downstream field evaluation phase rather than substituting for it [2,5]. Figure 3 presents a schematic model of this integration, identifying the phases of the conventional pipeline where SB offers the greatest time saving and those where field-based evaluation remains indispensable.

Under this integration model, line development can be conducted entirely under SB, from hybridisation to the final inbred generation, or partially—for example, by accelerating only the early segregating generations before transferring material to the field, or by performing hybridisation under conventional conditions while conducting the inbreeding phase under SB. Genomic selection combined with SB has been reported to shorten the wheat breeding cycle from twelve to seven years while increasing genetic gain relative to conventional phenotypic selection [27], a result broadly consistent with the cycle time reductions summarised by Chaudhary and Sandhu [8] and Potts et al. [7]. Such figures should nonetheless be read as best-case outcomes for traits well suited to controlled-environment selection; for yield and abiotic stress tolerance, the limiting step remains, and is likely to remain, multi-environment field testing.

#### 2.3.6. Complementary Roles of Artificial Intelligence and High-Throughput Phenotyping

Beyond its integration with MAS, GS, and genome editing, SB is increasingly discussed alongside artificial intelligence (AI) and automated, high-throughput phenotyping (HTP) as complementary accelerants of the breeding cycle. AI-enabled image analysis and machine learning models can extract morphological and physiological trait data from large SB-grown populations far faster than manual scoring, while automated platforms reduce the labour burden associated with the dense planting and frequent handling that SB requires [28]. More recent proposals envisage AI operating not only on phenotypic data but also directly on genomic and multi-omics information to guide parental selection and predict trait outcomes before a cross is made, a capability that would compound with SB’s shortened cycle time by allowing each accelerated generation to be informed by a progressively refined predictive model [29]. Realising this synergy in practice will depend on standardised, machine-readable phenotypic data capture within SB facilities, an infrastructure requirement that compounds the cost and equity concerns discussed in Section 2.3.3.

### 2.4. New Genomic Techniques Versus GMOs: Complementarity with Conventional and Speed Breeding

The regulatory transformation referenced in the Introduction—the European Parliament’s final approval on 17 June 2026 of the new genomic technique (NGT) regulation—makes it necessary to examine how NGT-derived plants differ fundamentally from genetically modified organisms (GMOs) and how these techniques can enhance, rather than supplant, conventional and speed breeding pipelines [6]. The distinction is not merely legislative but grounded in the nature of the genetic modification itself. Classical transgenic GMOs are characterised by the stable insertion of heterologous DNA sequences derived from sexually incompatible organisms into the host genome at a genomically non-specific locus, resulting in a chimeric genotype that could not arise through conventional hybridisation or natural mutation. New genomic techniques, by contrast—and, in particular, the CRISPR/Cas9 system (Clustered Regularly Interspaced Short Palindromic Repeats and its associated endonuclease Cas9)—achieve targeted, sequence-specific modifications of the endogenous genome without the permanent incorporation of foreign DNA into the final plant [30,31].

CRISPR/Cas9 operates by delivering a guide RNA that directs the Cas9 endonuclease to a predetermined genomic locus, where a double-strand break is introduced and subsequently resolved by the cell’s own DNA repair machinery; the resulting small insertions, deletions, or substitutions are indistinguishable, at the sequence level, from alleles arising through spontaneous or chemically induced mutagenesis that have long been deployed in conventional breeding without GMO oversight [32]. It is precisely this equivalence to naturally occurring variation that underpins the NGT regulation’s Category 1 classification: plants carrying modifications that could also have arisen through conventional breeding are, under the new framework, exempt from the requirements of GMO legislation [6,33]. The European Food Safety Authority confirmed in 2024 that Category 1 NGT plants present no additional hazards compared to products of conventional breeding [34].

Within a speed breeding context, the integration of CRISPR-based NGT approaches offers a particularly powerful acceleration of the breeding cycle: whereas introgression of a desired allele through successive backcross generations under speed breeding may still require three to five rapid cycles, a single CRISPR editing event can introduce an equivalent targeted mutation directly into an elite background in a single generation, after which SB is used to confirm homozygosity and advance the edited material through phenotypic and molecular evaluation. Strategies such as ‘ExpressEdit’, in which preassembled Cas9–sgRNA ribonucleoprotein complexes are delivered directly to shoot apical meristems to bypass tissue culture, pair naturally with the rapid generation turnover of speed breeding and have been reported to reduce the interval from target identification to homozygous, phenotyped lines to fewer than twelve months in wheat and barley [2]. The synergy extends to multiplex genome editing, where multiple loci are edited simultaneously in a single transformation event and the resulting segregating population is then advanced through SB, compressing what would otherwise be decades of sequential backcrossing and selection into a single integrated pipeline [35]. Critically, because NGT Category 1 plants are treated as conventionally bred varieties under the new EU framework, edited lines emerging from such pipelines can, once the regulation enters into force, access the standard variety registration pathway without the time and cost burden of GMO authorisation, potentially eliminating the largest regulatory bottleneck that has historically constrained the deployment of precision-edited germplasm in European variety development programmes [6,33].

## 3. Materials and Methods

This article is structured as a narrative literature review rather than a systematic review with quantitative meta-analysis, in keeping with the comparative and synthetic aim of the work. The primary source anchoring the technical sections is the protocol review of Blinkov et al. [5], which consolidates speed breeding factors and protocols for approximately thirty crop species. It was the principal reference framework against which other reviews and primary studies were compared and cross-checked.

Additional sources were identified through a combination of (i) the reference lists of the foundational speed breeding papers [4,14]; (ii) subsequent review articles published between 2020 and 2025 that synthesise speed breeding methodology and applications [2,7,8,9,13]; and (iii) the lead author’s own lecture materials on speed breeding prepared for teaching the course Plant Breeding and Crop Improvement within Recrop COST network, held at the Faculty of Agriculture, University of Novi Sad. Sources were selected for inclusion if they reported either (a) a validated, crop-specific speed breeding protocol with quantified generation time or generations per year, or (b) a structured comparison of speed breeding against conventional or other accelerated breeding methods. In total, 34 sources meeting these criteria were retained for synthesis, comprising eight principal review and protocol papers subjected to detailed comparative analysis (Table 1), together with additional primary studies, application case reports, and regulatory or policy documents cited throughout the text.

For the comparative synthesis presented in Table 1, each source was read in full and its reported quantitative parameters—photoperiod, light intensity, temperature regime, and achieved generations per year—were extracted and cross-tabulated by crop and by source. Where two sources reported different values for the same crop under nominally similar conditions, both values are retained in the table and the discrepancy is noted in the text, consistent with the observation that speed breeding efficacy is strongly genotype-dependent [5,36]. No new experimental data were generated for this review; all figures and the comparative table are derived from previously published results, redrawn and re-tabulated for clarity and consistency of units.

## 4. Conclusions

By simultaneously targeting photoperiod, light spectrum and intensity, temperature, carbon dioxide concentration, mineral nutrition, substrate volume and post-harvest seed dormancy, speed breeding addresses the dominant constraint on genetic gain in conventional plant breeding—the multi-year time requirement for producing homozygous, field-evaluable inbred lines—and compresses it from years to months. The evidence synthesised in this review confirms that well-validated protocols are now available for long-day cereals and legumes, capable of delivering four to seven generations per year, as well as for a growing range of short-day and day-neutral crops—soybean, rice, amaranth and hemp among them—for which tailored LED-based regimes support four to five generations per year.

Cross-reading Blinkov et al. [5], Watson et al. [4], Ghosh et al. [14], Jähne et al. [9], Pandey et al. [2], Chaudhary and Sandhu [8], Potts et al. [7] and Samantara et al. [13] reveals a strong shared foundation on the core environmental parameters of SB, while exposing genuine, unresolved disagreements regarding the generality of far-red light supplementation and the magnitude of generational gains achievable in different legume species. When SB is coupled with MAS, genomic selection and genome editing, its impact on genetic gain is considerably amplified, enabling early culling of off-type seedlings and shortening combined breeding-and-selection cycles that would otherwise span more than a decade. A concrete illustration is provided by the combination of genomic selection with SB, which has been shown to shorten the wheat breeding cycle from twelve to seven years while increasing realised genetic gain relative to conventional phenotypic selection [27]. Similarly, coupling SB with marker-assisted selection has enabled the targeted introgression of disease resistance and quality trait alleles, such as the hst1 salt tolerance gene in rice [19] and the Glu-B1i glutenin subunit in bread wheat [20,21], within a fraction of the time required by conventional backcrossing alone.

Nevertheless, the critical analysis presented here makes it clear that SB must not be positioned as a full substitute for conventional field-based selection. The G × E interaction effects that shape adaptive differentiation in target growing environments, the restricted set of traits that can be meaningfully evaluated under controlled-environment conditions, the considerable capital and energy demands that limit equitable global uptake, and unresolved questions concerning the biological integrity of rapidly advanced generations all argue for treating SB as one integrated element of a broader breeding strategy. Its greatest contribution lies in supplying well-characterised early-generation material to a downstream pipeline in which multi-environment field testing remains the decisive and irreplaceable final step before variety release. Future research should focus on the standardisation of protocols for a wider range of plant species, a reduction in energy costs associated with implementation, and a more comprehensive assessment of the long-term genetic and epigenetic consequences of accelerated breeding. In the context of the growing challenges posed by climate change and the need to improve global food security, speed breeding represents one of the most promising technologies for the development of new, higher-yielding and more resilient crops. In particular, integrative, polygenic traits such as yield and tolerance to abiotic stress cannot be reliably evaluated under SB, because their expression depends on genotype × environment interactions and field-level stress exposure that a uniform, optimised growth chamber environment does not reproduce (Section 2.3.1 and Section 2.3.2); such traits require confirmation in multi-environment field trials regardless of how efficiently early generations were advanced under SB.

## Figures and Tables

**Table 1:** Comparative summary of major speed breeding reviews and protocol papers: principal focus and key reported findings.

Source	Type	Principal Focus and Key Reported Findings	Generations/Year Reported
Blinkov et al. [5], Front. Plant Sci.	Review	Comprehensive systematisation of all factors (photoperiod, light spectrum/intensity, temperature, CO_2_, vernalisation, mineral nutrition, substrate volume, tiller removal, growth regulators) influencing SB across ~30 crops; consolidates embryo culture and forced drying protocols and genetic engineering approaches.	2–6 (crop-dependent); up to 7 in chickpea
Watson et al. [4], Nat. Plants	Primary/protocol	Founding demonstration of SB at scale; 22 h/2 h photoperiod with immature seed harvest; first report of 6 generations/year in wheat, barley, chickpea and pea, 4 in rapeseed; established growth chamber, glasshouse and low-cost-room implementations.	4–6
Ghosh et al. [14], Nat. Protoc.	Protocol	Detailed, step-by-step methodology accompanying Watson et al. [4]; specifies PAR range (400–700 nm), dawn/dusk simulation, and immature seed drying/germination procedures.	4–6
Jähne et al. [9], Theor. Appl. Genet.	Primary/protocol	First validated LED-controlled SB protocol for short-day crops; blue-enriched, far-red-deprived spectrum at 10 h photoperiod; demonstrates strong genotype- and species-specific response to far-red light (positive in rice/amaranth, neutral in soybean).	Soybean 5; rice and amaranth flowering accelerated 8–20 days by far-red light
Pandey et al. [2], Plant Breed.	Review	Frames SB as one of several accelerated strategies; emphasises integration with MAS, genomic selection, pollen-based selection and genome editing; tabulates SB applications across 19 crop species.	2–8 (crop-dependent)
Chaudhary and Sandhu [8], Euphytica	Review	Historical account tracing SB to 1980s USDA and NASA work; compiles list of SB-derived cultivar releases (2014–2021) and direct cost data (>50% of SB cost attributable to lighting/temperature control).	4–7.6 depending on crop and protocol
Potts et al. [7], Crops	Review	Traces SB lineage to 19th century carbon arc lamp experiments; situates SB alongside single-plant selection and SSD; reviews high-density planting as a low-cost acceleration strategy and surveys infrastructure barriers in developing countries.	4–7 (crop-dependent); up to 8 in cowpea
Samantara et al. [13], Biology	Review	Perspective review emphasising SB’s alignment with single-plant selection and SSD, and its capacity to exploit allelic diversity from landraces and wild progenitors; surveys applications in genetic mapping, genetic modification and trait stacking.	Crop-dependent

Note: generations/year figures are as reported in the cited source and reflect different genotype panels, facility specifications and harvest criteria; direct comparison across sources should account for these differences.

## Data Availability

No new data were created or analysed in this study. Data sharing is not applicable to this article.

## Abbreviations

AI, artificial intelligence; CO_2_, carbon dioxide; DH, doubled haploid; FHB, Fusarium head blight; GA, gibberellic acid; GEBV, genomic estimated breeding value; G × E, genotype-by-environment interaction; GS, genomic selection; HDP, high-density planting; HTP, high-throughput phenotyping; LED, light-emitting diode; MAS, marker-assisted selection; NGT, new genomic technique; PAR, photosynthetically active radiation; QTL, quantitative trait locus; RGA, rapid generation advance; SB, speed breeding; SSD, single seed descent.
